# An Exploratory Pathways Analysis of Temporal Changes Induced by Spinal Cord Injury in the Rat Bladder Wall: Insights on Remodeling and Inflammation

**DOI:** 10.1371/journal.pone.0005852

**Published:** 2009-06-09

**Authors:** Silvia Wognum, Claudio E. Lagoa, Jiro Nagatomi, Michael S. Sacks, Yoram Vodovotz

**Affiliations:** 1 Department of Bioengineering, University of Pittsburgh, Pittsburgh, Pennsylvania, United States of America; 2 The McGowan Institute, Pittsburgh, Pennsylvania, United States of America; 3 Department of Surgery, University of Pittsburgh, Pittsburgh, Pennsylvania, United States of America; 4 Center for Inflammation and Regenerative Modeling, The McGowan Institute, University of Pittsburgh, Pittsburgh, Pennsylvania, United States of America; Virginia Tech, United States of America

## Abstract

**Background:**

Spinal cord injuries (SCI) can lead to severe bladder pathologies associated with inflammation, fibrosis, and increased susceptibility to urinary tract infections. We sought to characterize the complex pathways of remodeling, inflammation, and infection in the urinary bladder at the level of the transcriptome in a rat model of SCI, using pathways analysis bioinformatics.

**Methodology/Principal Findings:**

Experimental data were obtained from the study of Nagatomi *et al*. (Biochem Biophys Res Commun 334: 1159). In this study, bladders from rats subjected to surgical SCI were obtained at 3, 7 or 25 days post-surgery, and Affymetrix GeneChip® Rat Genome U34A arrays were used for cRNA hybridizations. In the present study, Ingenuity Pathways Analysis (Ingenuity® Systems, www.ingenuity.com) of differentially expressed genes was performed. Analysis of focus genes in networks, functional analysis, and canonical pathway analysis reinforced our previous findings related to the presence of up-regulated genes involved in tissue remodeling, such as lysyl oxidase, tropoelastin, TGF-β1, and IGF-1. This analysis also highlighted a central role for inflammation and infection, evidenced by networks containing genes such as CD74, S100A9, and THY1.

**Conclusions/Significance:**

Our findings suggest that tissue remodeling, infection, inflammation, and tissue damage/dysfunction all play a role in the urinary bladder, in the complex response to SCI.

## Introduction

Spinal cord injuries (SCI) rostral to the lumbar spine can lead to severe urinary tract dysfunctions in humans, including bladder areflexia, hyperreflexia, and detrusor-sphincter dyssynergia [1]. In addition to functional deficiencies, these bladder pathologies involve changes in tissue morphology such as hypertrophy [2] and fibrosis [3], as well as significant changes in mechanical properties [4]. Specifically, chronic neurogenic bladders tend to be less compliant than normal bladders [3]–[5]. This loss of compliance appears to be related to changes in composition of collagen type I and III within the detrusor tissue, shown by increased ratios of collagen type III/collagen type I mRNA transcripts within the detrusor tissue [3].

We have previously utilized a rat model of SCI to investigate the temporal changes in tissue composition and mechanical properties in the urinary bladder. We demonstrated that the acute response of the rat bladder wall tissue (in the first 10 days following injury), included becoming thicker, and significantly more compliant [6]. In addition, biochemical assays revealed that while the relative collagen concentration decreased, the relative elastin concentration of the SCI bladder was significantly greater compared to normal bladders [7]. Recent results show that the long-term response (up to 10 weeks after injury) differed from the acute response, i.e. bladder wall compliance was found to be significantly greater at 3 and 6 weeks post-SCI when compared to the normal bladders, but at 10 weeks compliance substantially reduced to near that of normal bladders [8]. This trend in mechanical compliance closely paralleled the collagen/elastin ratio. This study showed that while there are some similarities between acute and chronic responses of the urinary bladder wall tissue to SCI, the overall alterations are distinct. Furthermore, this study explained the discrepancies between the clinical finding of low tissue compliance and our earlier experimental finding of high compliance [8]. Other acute remodeling evidence included smooth muscle cell hypertrophy and changes in smooth muscle bundle orientation from the longitudinally biased orientation in the normal bladders to the biphasic (both longitudinal and circumferential) orientation in the SCI bladders [9]. The molecular mechanisms underlying the observed changes in tissue composition, including the increase in elastin content, in the bladder following SCI have not yet been elucidated. A first step in this was taken by our laboratory by performing gene microarray analysis and real-time q-PCR on rat bladders at various time points following SCI [10]. The study revealed that the mRNA levels for tropoelastin and lysyl oxidase were as much as eight-fold higher in the bladders of SCI rats as compared to control animals. This study also demonstrated that mRNA and protein levels of the cytokines transforming growth factor-β1 (TGF-β1) and insulin-like growth factor-1 (IGF-1), known stimulators of elastin synthesis [11]–[14], were significantly higher in SCI bladders as compared to those of normal rats. Taken together, these results suggested that changes in the mechanical environment of the bladder trigger synthesis of soluble growth factors by the bladder cells, which induce downstream tissue remodeling.

The tissue responses to injury and stress are complex and multi-faceted, and therefore important information can be lost if these responses are examined in a reductionist fashion, i.e., one gene at a time. An alternative approach is to examine the response of the system as a whole, and gene microarray analysis has emerged as a useful tool with which to interrogate numerous signaling pathways and biological processes together. To facilitate the analysis of the gene microarray data, and to relate gene up- and down-regulation to underlying biological processes, various groups have proposed using *in silico* genomics network analysis [15]–[19], one variant of which is Ingenuity Pathways Analysis (IPA) (Ingenuity® Systems, www.ingenuity.com).

In the present study, microarray data of bladders from SCI rats, at different time points after injury, were analyzed using IPA. Relevant molecular networks and functions that are involved in the response of bladder tissue to external stress were explored and modulated. Our findings suggest that tissue remodeling, infection, inflammation, and damage/dysfunction all play a role in the complex response to SCI.

## Methods

### Ethics statement

Data from urinary bladders of rats with experimental SCI were used in this study. We have chosen the smallest animal species that is generally accepted as an appropriate model for SCI. Following SCI, rat bladders show similar behavior to those of humans, and any phylogenetically lower species would be difficult to simulate human SCI. All animals were treated and cared for in accordance with our animal use protocol approved by the Institutional Animal Care and Use Committee (IACUC) of the University of Pittsburgh. Spinalized rats were kept in a single occupancy cage in a climate-controlled facility. The bladders of the spinalized rats were expressed two times a day to relieve the animals from overfilling for the first two weeks following surgery or until reflex voiding emerged. Both laminectomy and bladder harvesting were performed under halothane anesthesia.

### Preparation of samples and microarray analysis

As described in our previous study [10], female Sprague-Dawley rats (170 g) underwent a complete laminectomy at the T9-T10 spinal level, under halothane anesthesia, according to established protocols [20]. Bladders were harvested from one normal and three SCI rats (one each; 3, 7 or 25 days post-surgery) under halothane anesthesia. The muscle layer of each sample was dissected, snap-frozen in liquid nitrogen and stored until further processing.

Total RNA was isolated using Trizol® reagent, and following the manufacturer's instructions (Invitrogen, Carlsbad, CA). Preparation of cRNA, hybridization, and scanning of microarrays was performed according to established methods [21], following the manufacturer's protocols (Affymetrix, Santa Clara, CA). Gene transcripts expressed by the cells from one bladder in each group (normal, 3 days, 7 days, and 25 days) were analyzed and compared using Affymetrix GeneChip® Rat Genome U34A array (Affymetrix, Santa Clara, CA). The chips were scanned in a Hewlett-Packard ChipScanner to detect hybridization signals and the data were analyzed using Affymetrix GeneChip® Analysis Suite Software. Statistical tests performed on the data included determining if genes were significantly present or absent in the sample (*p*<0.05), as well as a comparison analysis between experiment vs. baseline. Gene transcripts for the normal rat bladder were used as the control for comparison with SCI groups to determine the change in transcription levels from this baseline, and if the change could be considered significant, based on a *p-*value of the change. A quantitative estimate of the change in gene expression was given by the signal log ratio, and the fold increase was calculated as fold increase = 2^(log ratio)^. The microarray data have been deposited in NCBI's Gene Expression Omnibus [22] and are accessible through GEO Series accession number GSE14096.

Genes that were regulated at least 3.0-fold (both up and down) were considered significant as in our previous study, and were used for further analysis with IPA, irrespective of the present/absent call. In addition, to reduce the number of genes to analyze, a second measure was used as suggested by IPA. In this method, genes considered by the software to have not changed significantly were discarded from the data set based on the change *p-*value, which ranges in scale from 0.0 to 1.0. Values close to 0.0 indicate likelihood for an increase in transcript expression level as compared to baseline, and values close to 1.0 indicate likelihood for a decrease. An increase in transcription level was considered significant at a *p-*value of 0.003 or lower, while a significant decrease was considered at a *p-*value of 0.997 or higher.

### Ingenuity Pathways Analysis

The web-based pathways analysis tool IPA (Ingenuity Systems®, www.ingenuity.com) was used to identify biological and molecular networks underlying bladder tissue remodeling after SCI. This web-based entry tool allows for the mapping of gene expression data into relevant pathways based on their functional annotation and known molecular interactions [23]–[25]. This knowledge coming from published, peer-reviewed scientific publications is stored in the Ingenuity Pathways Knowledge Base (IPKB), and is continuously updated. A molecular network of direct or indirect physical, transcriptional, and enzymatic interactions between mammalian orthologs was computed from this knowledge base. By comparing the imported microarray data with the IPKB, the list of genes was transformed in a set of relevant networks, focus genes and canonical pathways were identified, and functional annotation was performed.

The genes considered to have been differentially regulated to a significant extent were uploaded into IPA along with the gene identifiers and corresponding fold change values. Each gene identifier was mapped to its corresponding gene object in the IPKB. The genes from the data set were overlaid onto a global molecular network developed from information contained in the IPKB. In the *network analysis*, networks of these genes are then algorithmically generated based on their connectivity. Two genes are considered to be connected if there is a path in the network between them. Highly-interconnected networks likely represent significant biological function, which is defined by an optimized triangular relationship. Focus genes are defined based on triangular connectivity, and they are ranked in decreasing order of triangular connectivity. IPA constructs networks that optimize for both interconnectivity and number of focus genes under the constraint of maximal network size.

In the graphical representation of a network, genes or gene products are represented as nodes, and the biological relationship between two nodes is represented as an edge (line). All edges are supported by at least one reference as stored in the IPKB. Human, mouse, and rat orthologs of a gene are stored as separate objects in the IPKB, but are represented as a single node in the network. The intensity of the node color indicates the degree of up- (red) or down- (green) regulation of a given gene. Nodes are displayed using various shapes that represent the functional class of the gene product. Edges are displayed with various labels that describe the nature of the relationship between the nodes.

The *functional analysis of a network* identified the biological functions and/or diseases that were most significant to the genes in the network, and *the functional analysis of the entire data set* identified the biological functions and/or diseases that were most significant to the data set. The network genes or the genes for the data set, that were associated with biological functions and/or diseases in the IPKB were considered for the analysis. Fischer's exact test was used to calculate a *p-*value determining the probability that each biological function and/or disease assigned to that network or to the data set is due to chance alone.

*Canonical pathways analysis* identified molecular pathways from the IPA library of canonical pathways (part of the IPKB) that were most significant to the data set. Genes from the data set that were associated with a canonical pathway in the IPKB were considered for the analysis. The significance of the association between the genes from the dataset and the canonical pathway (in the IPKB) was measured in two ways as described in IPA documentation: 1) A ratio was calculated of the number of genes from the dataset in a given pathway divided by the total number of molecules that make up the canonical pathway; 2) Fisher's exact test was used to calculate a *p-*value determining the probability that there is an association between the genes in the dataset and the canonical pathway that cannot be explained by chance alone.

In the present study, only the networks with the highest score, and which contained relevant genes as based on our previous study [10] were selected for analysis. They were subsequently merged to form a single network representing the major underlying biology of the process. This was followed by functional analysis on the data set level, and canonical pathway analysis.

## Results

### DNA microarray results

As described in the previous study by Nagatomi *et al*. [10], gene array analysis of bladder mRNA tissue revealed that transcription levels for over 100 genes in SCI rats were either increased or decreased at least 3-fold as compared to those of normal rats. In that prior analysis, special attention was paid to select genes encoding the extracellular matrix proteins, growth factors linked to structural protein synthesis, and smooth muscle hypertrophy and hyperplasia. The expression of tropoelastin and lysyl oxidase mRNA were 8- and 6-fold higher, respectively, in SCI rats at 3 days as compared to normal rats. At day 7 and 25 the levels decreased, but they remained at least 3-fold higher than in normal rats. In addition, mRNA levels for TGF-β1 and IGF-1 in SCI rats increased to 13- and 5-fold levels respectively, at 3 days post-injury. IGF-1 levels decreased after this initial up-regulation, but stayed above 3-fold, whereas TGF- β1 levels stayed as high as 9-fold at both day 7 and day 25.

Up-regulation of tropoelastin and lysyl oxidase was further confirmed and quantified at the mRNA level using total RNA collected from three to four bladders each of normal and post-SCI (3 days, 7 days, and 25 days) rats and TaqMan analysis according to the standard protocols with the Mx3000P qPCR apparatus (Stratagene, La Jolla, CA), as described previously [10]. mRNA expression for tropoelastin and lysyl oxidase was found to be significantly higher at 3 and 7 days post-SCI as compared to normal, and returned to normal levels at 25 days. Also, concentrations of TGF-β1 and IGF-1 in the bladder protein samples were quantified using a commercially available immunoassay kit and following manufacturer's instructions, as described in [10]. This analysis confirmed that both transcription and translation were up-regulated as a result of SCI.

In our previous study, gene expression arrays were utilized as a first pass to identify potential target genes. The present study further explores those datasets using IPA.

### Genes and gene networks induced post-SCI

**Figure 1:**
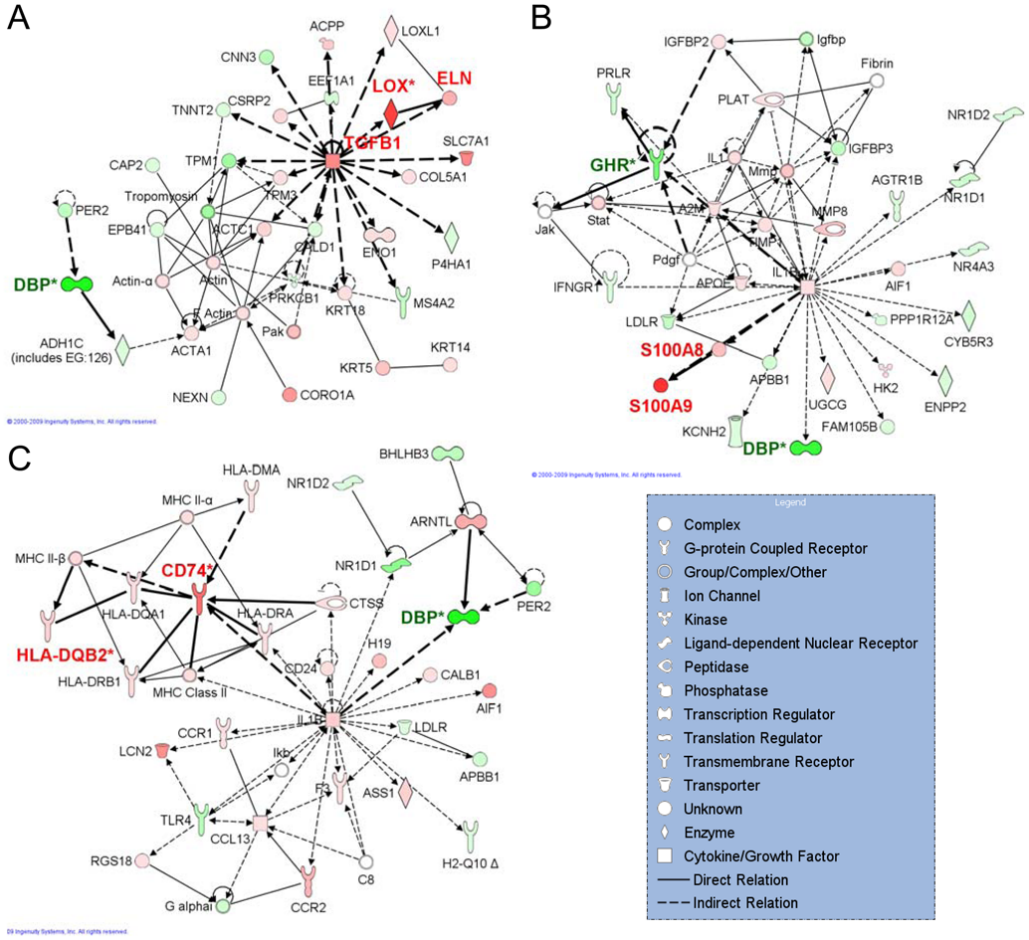
A single network for each time point post-SCI showing relevant genes. A) The first network of day 3 post-SCI with a maximum score of 30. This network clearly shows the direct relation between elastin and LOX, and an indirect relation between TGF-β1 and elastin. B) The second network of day 7 post-SCI showing the highly up-regulated damage-associated genes S100A8 and S100A9. C) The second network of day 25 post-SCI showing the immune response associated genes CD74 and HLA-DQB2. An asterisk (*) indicates that a given gene is represented in the microarray set with multiple identifiers.

The microarray data were analyzed using IPA for all three time points, comparing SCI rats to a non-manipulated control. From each data set, 558 (day 3), 361 (day 7), and 395 (day 25) genes, respectively, were deemed to be expressed differentially and were uploaded into IPA accordingly. We first examined the most relevant focus genes and their respective networks (score >27). The most significantly evoked networks for each time point post-SCI, including the top biological functions underlying the networks, can be found in the Supplementary Tables S1, S2, S3. To identify all relevant genes based on both the fold-change and the network score, the most highly-scored networks for each time point were merged and investigated separately (See Supplementary Figures S1, S2, S3). At day 3, the networks that received the highest scores (seven total) suggested important changes in gene expression. Tables 1–2 show a summary of all genes that were found to be of interest based on their fold change value, or on their central location. The numbers can also be found in Figure 1, where one network from each time point is shown, emphasizing important genes. These networks represent examples of remodeling, damage, and inflammation/infection, respectively.

**Table 1:** List of relevant genes up-regulated by SCI.

ID	No.	Gene name	Gene description and relevant information	Fold change Day 3	Fold change Day 7	Fold change Day 25
S66184_s_at, rc_AA875582_at	1	LOX*	lysyl oxidase, crosslinking of collagens and elastins	21.1	3.5	
J04035_at	2	ELN	tropoelastin	8.6	3.0	3.5
X52498cds_at	3	TGFβ1	involved in multiple functions and diseases	13.0	9.2	9.2
X13044(cds)_(g)_at	4	CD74*	cytokine binding, major histocompatibility complex (MHC) class II molecule, antigen presentation	21.1	12.1	26.0
U65217_i_at X56596_at	5	HLA-DQB2*	major histocompatibility complex (MHC) class II molecule, antigen presentation	19.7	14.9	8.0
L18948_at	6	S100A9	damage-associated molecular pattern (DAMP) molecule	9.2	207.9	194
rc_AA957003_at	7	S100A8	damage-associated molecular pattern (DAMP) molecule		14.9	13.0
X02002_at	8	THY1	surface receptor on T-lymphocytes	19.7	24.3	7.0
S74351_s_at S81478_s_at U02553cds_s_at	9	DUSP1*	involved in inflammatory response	17.1		9.8
M65149_at rc_AI045030_s_at	10	CEBPD	involved in inflammatory and immune responses	14.9	9.2	4.9
M24067_at	11	Serpine1	serum protease inhibitor, involved in may pathological processes	13.9	4.3	
D00698_s_at	12	IGF1	involved in multiple processes and diseases	4.9	3.7	
M29866_s_at	13	C3	activation of complement system, immune response to bacteria		19.7	36.8

* Gene is represented in the microarray set with multiple identifiers.

**Table 2:** List of relevant genes down-regulated by SCI.

ID	No.	Gene name	Gene description and relevant information	Fold change Day 3	Fold change Day 7	Fold change Day 25
J03179_(g)_at	14	DBP*	D site of albumin promoter binding protein, transcription activator and regulator	−45.3	−29.9	−55.7
Z83757mRNA_(g)_at	15	GHR*	growth hormone receptor		−21.1	−4.9
J03819_at	16	THRB	thyroid hormone receptor beta	−59.7	−34.3	
AF007836_at	17	RIMS1	synaptic vesicle protein, regulating synaptic membrane exocytosis	−39.4		
X51529_at	18	PLA2G2A	phospholipase A2, group IIA	−34.3		
U17837cds_at	19	PRDM2	PR domain containing 2, transcription regulator, tumor suppressor gene		−45.3	
X53087cds_at	20	IL4	interleukin-4, cytokine, immune response		−16.0	
M31837_at, rc_AI009405_s_at	21	IGFBP3*	insulin-like growth factor binding protein 3			−34.3

* Gene is represented in the microarray set with multiple identifiers.

### Evidence for remodeling post-SCI based on gene network analysis

The importance of the molecules that are important in remodeling, i.e. lysyl oxidase (LOX), tropoelastin (ELN), TGF-β1, and IGF-1, as established by Nagatomi *et al*. [10] were confirmed in the present study. TGF-β1 and IGF-1 both play prominent roles in many cellular processes, including tissue remodeling, growth, and development. They both occupy a central position in the networks, and hence are important players in the pathological process, although their expression (in fold-change) was not as high as compared to some other molecules (Table 1). IGF-1 is a very central molecule at day 3 and day 7 post-SCI, but it disappeared at day 25. The most significant network at day 3 post-SCI, with 30 focus genes and a score of 49, suggests an indirect or not yet established relation between TGF-β1 and tropoelastin (Figure 1a). A previous study found that human TGF-β1 protein increases the activity of rat LOX protein in ovary granulosa cells in a dose-dependent manner [26], but this relationship has not been explored in the bladder or in the setting of SCI. TGF-β1 has also been shown to have significant effect on collagen organization in collagen gels seeded with bladder smooth muscle cells [27], [28].

The network analysis confirms the up-regulation of tropoelastin following SCI as compared to normal tissue, though this up-regulation does not occur to a very large extent as compared to other transcripts (notice the light red color in Figure 1). Our previous studies have confirmed the significance of increased elastin concentrations in rat bladders at different time-points after SCI [8], [29]. Tropoelastin appears in the most significant networks at day 3 and 7, but not at day 25. At day 25, tropoelastin appears in network 11, which contains mainly the following functions: “Cellular growth and proliferation”, “Cell cycle”, and “Cellular movement”. In this network, complement component 3 (C3), which plays a central role in the activation of complement system, is the most prominent molecule, with a fold change of 36.8. Recently, it has been established that C3 plays an important role in the inflammation and secondary injury of the spinal cord following SCI, although little is still known about its exact role [30].

The protein encoded by the LOX gene is an extracellular copper-containing enzyme that initiates the cross-linking of collagens and elastin. In the networks, LOX shows a direct relation with elastin (Figure 1), but in addition, it has been shown to be involved in the organization of collagen fibrils [31]. Over-expression of LOX in a rat dermal wound healing model resulted in significantly enhanced mechanical strength of the wound site, indicating increased cross-linking of the extracellular matrix[31].

### Evidence for inflammation post-SCI from gene network analysis

Nagatomi *et al*. focused entirely on tissue remodeling and the genes involved in that process [10]. The present IPA analysis revealed that processes that are related to inflammation and infection are also highly prevalent in the data sets, especially at 25 days post-SCI. This led to our additional focus on the networks that showed highly up-regulated genes that are involved in inflammation and infection.

The most remarkable sign of tissue damage and inflammation is the presence of S100A9 (S100 calcium binding protein A9) as early as day 3, but with fold change values around 200 at day 7 and day 25 (Table 1, Figure 1b). Phagocytic S100 proteins are calcium binding proteins that are a well-known example of damage-associated molecular pattern (DAMP) molecules, which mediate inflammatory responses and recruit inflammatory cells to sites of tissue damage [32]. S100A8 (also named calgranulin A; myeloid-related protein 8, MRP8), and S100A9 (calgranulin B; MRP14) are found in granulocytes, monocytes, and early differentiation stages of macrophages. They are found at high concentrations in inflamed tissue, where neutrophils and monocytes belong to the most abundant cell types. They exhibit pro-inflammatory effects *in vitro* at concentrations found at sites of inflammation *in vivo*. Expression of these proteins can also be induced in keratinocytes and epithelial cells under inflammatory conditions. Secretion of S100A8/S100A9 is induced during contact of phagocytes with inflamed endothelium [32].

The high up-regulation of CD74, HLA-DQB, and THY1 (Table 1, Figure 1c) suggests infiltration of inflammation-related cells, such as T lymphocytes and B lymphocytes, monocytes, and macrophages. As found from IPKB, CD74 and HLA-DQB2 are major histocompatibility complex (MHC) class II molecules. They are associated with the cell surfaces of B and T lymphocytes. CD74's main molecular function is cytokine binding, and both CD74 and HLA-DQB are involved in the antigen presentation canonical pathway and in the immune response. CD74 is shown to be present in the rat urothelium (the endothelial lining of the urinary bladder) [33]. CD74 bladder immunostaining, and total amount of bladder CD74 protein and mRNA, are increased in an acute model of bladder inflammation [33]. THY1 is a surface receptor on T-lymphocytes. Interestingly, THY1 is also involved in regulating collagen type I, smooth muscle alpha actin, and TGF-β1 in the renal glomerulus from rats exhibiting glomerulonephritis [34].

DUSP1 (dual specificity phosphatase 1, synonym: MKP-1) is highly up-regulated at day 3 and day 25 (Table 1). It belongs to the MAPK (mitogen-activated protein kinase) phosphatase family, and dephosphorylates and inactivates MAPKs. It is involved in various signal transduction mechanisms and is known to be part of the inflammatory response. DUSP1 is suggested to play an important role in the human cellular response to environmental stress, as well as in the negative regulation of cellular proliferation. The activation of DUSP1 seems to counterbalance the inflammatory response to PAR (protease-activated receptors) activation by avoiding prolonged activation of p38 MAPK and increased cytokine production in the rat urinary bladder, which was revealed using IPA [35]. Studies using cultured immortalized macrophages provided compelling evidence indicating that MKP-1 deactivates p38 and JNK, thereby limiting pro-inflammatory cytokine biosynthesis in innate immune cells exposed to microbial components; this effect was confirmed *in vivo* using MKP-1-deficient mice [36]. DUSP1 appears to be a major feedback regulator of the innate immune response, and to play a critical role in preventing septic shock and multi-organ dysfunction during pathogenic infection [36]. Importantly, DUSP1 increases migration of smooth muscle cells to soluble collagen protein(s) through chemotaxis [37], and hence is also involved in the remodeling process.

Serpine1 is highly up-regulated at day 3, but decreases after that. Serpine1 is a type of serine protease inhibitor (serine protease inhibitor clade E, member 1; also called plasminogen activator inhibitor 1, PAI-1), a type of enzyme that plays a central role in various pathological processes including coagulation, fibrinolysis, malignancy, and inflammation [38], [39]. In several *in vitro* studies, Serpine1 has been shown to regulate, and to be regulated by TGF-β1, and thus its elevated expression in our study may be a further indicator of increased TGF-β1 expression. More specifically related to the urinary tract and fibrosis, both Serpine1 and TGF-β1 are involved in diabetic nephropathy [40], and in the context of smooth muscle cells, TGF-β1 protein is involved in expression of human Serpine1 mRNA in smooth muscle cells from myometrium of human females [41].

Another important gene at day 3 is CEBPD. The protein encoded by the CEBPD gene (Table 1) (CCAAT/enhancer binding protein (C/EBP), delta) is a transcription factor which is important in the regulation of genes involved in immune and inflammatory responses [42].

### Down-regulated genes

In addition to the up-regulated genes, the analysis shows some remarkable, high fold-change values for down-regulated genes (Figure 1, Table 2). However, most of these genes are unrelated to inflammation or remodeling. DBP (D site of albumin promoter binding protein), a transcription activator and regulator, is the only gene that is down-regulated at all three time-points (Figure 1, Table 2). THRB (thyroid hormone receptor beta) is down-regulated at day 3 and 7 post-SCI, and GHR (growth hormone receptor) at day 7 (Figure 1b) and 25. Down-regulation of GHR implies that growth and proliferation are inhibited, and that apoptosis and cell death are prevalent. The cytokine interleukin 4 (IL-4) is also down-regulated. IL-4 is produced by activated T cells, and is involved in inflammation, fibrosis, and multiple immune diseases. The down-regulation of IL-4, a canonical Th2 cytokine, suggests that Th1 pathways might predominate in this setting. For completeness, Table 2 shows the relevant genes with high negative fold-change values.

### Functions

**Figure 2:**
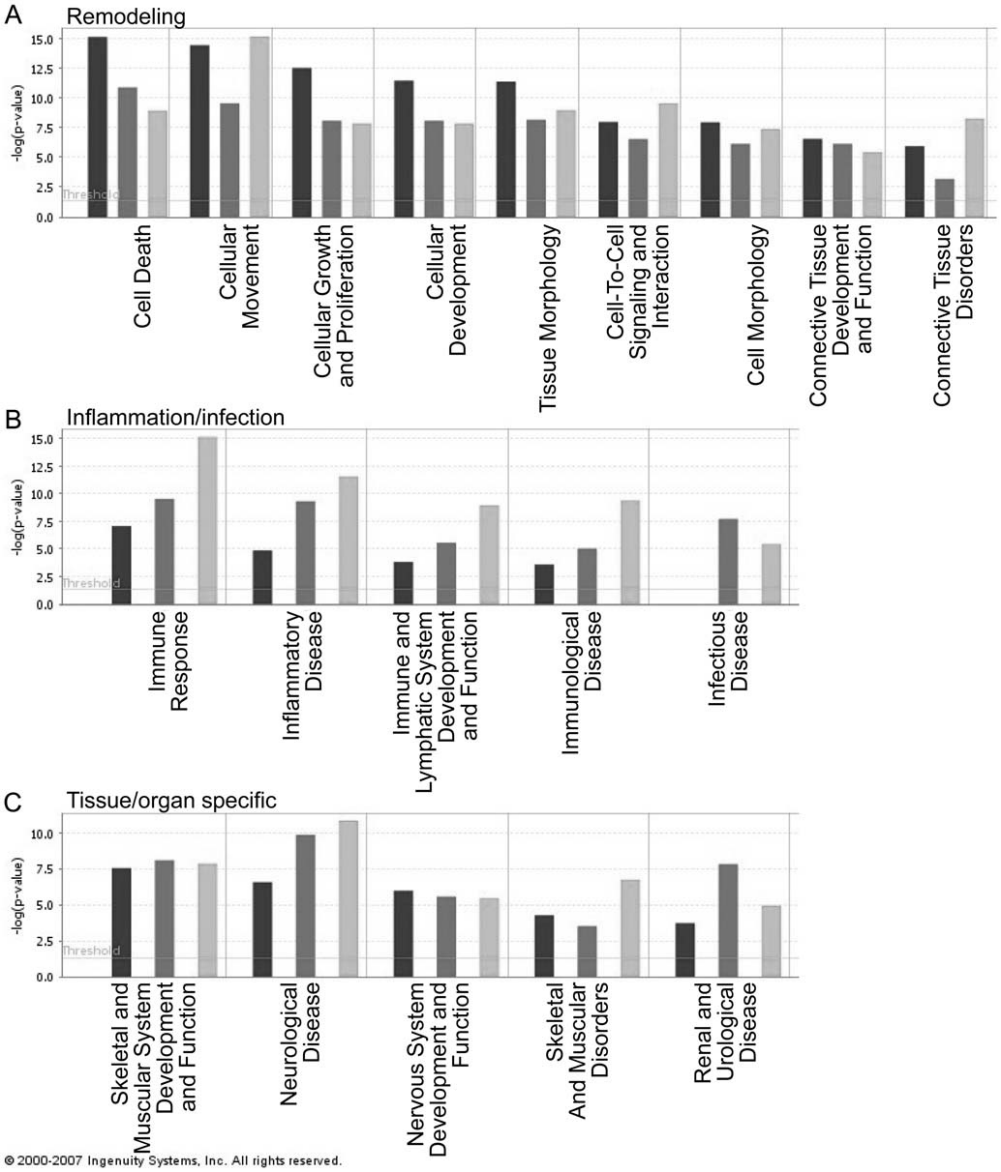
Functional analysis of all time points combined. Functions are listed by class: A) Functions related to remodeling; B) Functions related to inflammation, tissue damage, and infection; C) Bladder- or SCI-specific functions. The negative value of the log of the *p-*value is plotted for each function. Black is day 3, dark gray is day 10, and light gray is day 25.

Functional analysis of the three time points in the data set together reveals three important classes of functions, i.e. functions that are related to (1) remodeling, (2) inflammation, and tissue damage, (3) infection, and (4) bladder- or SCI-specific functions (Figure 2). More specifically, at day 3 after injury, remodeling and cellular processes prevail (Figure 2a), whereas at day 7 neurological disease, renal and urological disease, and inflammatory disease and immune response functions are initiated (Figure 2b–c). At day 25, inflammatory disease and immune response transcripts appear to be highly important (Figure 2c). The important functions, organized per time point and highlighting the important ones, are shown in Supplementary Figure S4.

For each function, we delved deeper into the specific genes that were involved, focusing on relevant cell types such as fibroblasts and smooth muscle cells. At all time points, DUSP1, IGF1, TGF-β1, Serpine1 are involved in growth, size and proliferation of connective tissue cells and (smooth) muscle cells. At 25 days, DUSP1 and IGF1 are involved in cell death and apoptosis of kidney cells, and failure and damage of the kidney. Also at the 25 day time point, the injured bladder tissue appears to undergo extensive activation, infiltration, and migration of inflammatory cells (“immune response”). Additionally, tropoelastin is involved in proliferation of muscle cells at day 3 and 7. Chemotaxis of monocytes and lysyl oxidase are involved in growth and proliferation of fibroblast cell lines at 3 days.

### Canonical pathways

At 3 days post-SCI, canonical pathways that involve synaptic processes are highly prevalent, presumably highlighting the effect of spinal cord transection on the nervous processes in the bladder. IGF signaling is present at all three time points. At day 7, pathways that are associated with inflammation appear, and at day 25, in addition to pathways associated with inflammation, those that suggest infection are visible. The canonical pathways for each time point are shown in Supplementary Figure S5.

## Discussion

### Significance and relevance of bladder inflammation after SCI

It is well-established that inflammation is a central driver of the physiology of people with spinal cord injuries, and it is apparent that inflammation has both beneficial and detrimental functions [43]–[45]. Traumatic central nervous system (CNS) injury can trigger severe systemic effects that lead to pathological autoimmunity, including the activation of T and B lymphocytes [45]–[48]. The bladder appears to be one of the organs that suffer from these systemic effects, as indicated by the present pathway analysis results. Deveaud *et al*. demonstrated that both lymphatic nodules and diffuse aggregates of lymphocytes were present in the lamina propria layers of many bladder tissue specimens, indicating inflammation and/or infection of the tissue [3]. This inflammation and infection are attributed to urine retention and catheterization. Inflammation is further related to connective tissue remodeling; the primary lesion or stimulus for alteration of connective tissue expression is not inflammatory, but in patients with conditions involving recurrent or chronic urinary tract infections and inflammation, there could be a secondary or additive effect due to cytokine expression.

Our present study suggests an important role for inflammation in the bladder after experimental SCI in the absence of known bacterial infection, perhaps related to direct injury to the spinal cord. The temporal process of inflammation and the differences between time points could be explained by differences in urine expression. The bladders were manually expressed twice daily up to two weeks post-injury, until the moment that reflex voiding started. In the first period, inflammation could be a result of bladder over-distension due to overfilling, whereas by the 25 day time point, hyper-contraction could be responsible for inducing a different inflammatory cascade. Few studies have been performed on the relationship between inflammation and the urinary bladder after SCI, except for inflammation as a result of bacterial infection. Results from a study on urinary bladder biopsies from SCI patients demonstrated no correlation between the number of bladder infections per year and the histopathology of the urinary bladder mucosa. In this study, 67.0% of the patients showed a chronic type of inflammation, and 24.5% showed a sub-acute type of infection, whereas all patients were free of clinical signs of infections. The inflammatory cell infiltrate was shown to be located solely in the lamina propria, together with fibrosis [49]. This study corroborates our results, which suggest that a different source for inflammation than bacterial infection alone must exist.

### Double identifiers

From the IPA analysis, we found a much higher up-regulation of lysyl oxidase than that reported in our previous study [10]. This transcript was elevated as much as 21 fold as compared to normal bladder at 3 days post-SCI, whereas a value of 8.5 was reported previously. A closer look into the data set revealed that LOX was represented in the microarray set with two identifiers (“S66184_s_at” and “rc_AA875582_at”, Table 1). For day 7 and day 25, the analysis showed the same extent of up-regulation as before, i.e. 3.5 and 2.8 respectively (S66184_s_at), and no significant up-regulation for the other identifier (rc_AA875582_at). In addition to unique probe sets, the Affymetrix GeneChip® consists of non-unique probe sets that recognize multiple alternative transcripts from the same gene (probe set type, “_a”), and probe sets with common probes among multiple transcripts from separate genes (“_s” suffix) (Affymetrix, ‘Array Design and Performance of the GeneChip® Rat Expression Set 230’). All genes with double identifiers are indicated in Table 1–2 with an asterisk.

### Significance of IPA

The sheer volume of data generated in typical microarray experiments often thwarts attempts at discerning central drivers of a given process. Ingenuity Pathways Analysis and similar *in silico* analysis tools have the potential to identify mechanisms behind experimental observations of changes in gene expressions, in different organ systems. To our knowledge, the present study is the first to elucidate genetic pathways in neuropathic bladders. Other urological applications involve the role of PARs in cystitis [35] (as described above), neuropathic bladder smooth muscle cells in culture [50], and diabetic bladders [51]. Dozmorov *et al*. [50] sought to identify candidate genes and pathways that are associated with increased thickness of smooth muscle layers observed in neuropathic bladder and the elevated cell proliferation observed in cultured neuropathic bladder smooth muscle cells as compared to their normal counterparts. Their analysis, based on a small set of 16 genes, identified the FGF, PTEN, and integrin signaling pathways to be involved in regulating bladder smooth muscle cell growth and proliferation and in the pathological development of the neuropathic cells. Hipp *et al*. [51] carried out pathways-based analysis, using a different technique (Expression Analysis Systematic Explorer, EASE, free software), of gene expression changes in urogenital smooth muscle from diabetic animals before the onset of physiologically significant diabetic complications, to identify and define diabetes induced, organ-specific smooth muscle responses. Interestingly, they found up-regulation of various cytokines and inflammatory mediators in both bladder and corporal smooth muscle tissue after experimentally induced diabetes. Diabetes, like SCI, is a systemic disorder that is not initiated in the bladder but that significantly affects this organ.

### Limitations

The findings presented herein must be evaluated in the context of several limitations. Our method of sorting the data and eliminating genes from the data might have increased the risk of including false positives in the final data set that was uploaded to IPA. The present/absent call was ignored because of the low n-number, and because the level of statistical significance is not necessarily related to the level of biological significance. The commonly accepted and generally used cut-off fold-change value of 2 [25] was increased to 3 to decrease the number of genes to be analyzed and to decrease the likelihood of possible false-positives.

Further limitations are related to the IPKB. It is a manually curated database that draws on the scientific literature, and the attribution of functions to gene sets is therefore somewhat subjective. As the literature evolves, so are the attributed pathways likely to evolve. The information in the IPKB, and the relations found in this study are not specific for bladder tissue, or for a smooth muscle containing tissue in general. However, the analysis gives a good indication of relevant processes by drawing parallels with knowledge obtained from other organ systems and *in vitro* studies. In addition, the information that is specifically found for smooth muscle cells, relates to vascular smooth muscle cells, which are known to differ from bladder smooth muscle cells in different aspects.

Another limitation in our study is that the tissue used for the gene array analysis contained other cell types in addition to smooth muscle cells, such as neural cells. In this study, it was impossible to distinguish between effects coming from the different tissue types, though the changes in expression of genes characterized as coming from an inflammatory infiltrate likely delineate the influx of such cells into the bladder. Some studies have carried out laser capture microdissection in order to obtain more focused tissue sources [52], but our intent was to examine the overall response of the bladder to SCI.

The present study did not validate the IPA results by q-PCR or immunoassays. However, the presence of tropoelastin and lysyl oxidase at the mRNA level, and TGF-β1 and IGF-1 at the protein level was quantified in our previous study [10]. We envision that the present results will be used as a guideline for future studies, and q-PCR or immunoassays should be performed before further conclusions are drawn from them.

### Conclusions

The present study follows up on earlier results that showed that several genes related to structural protein synthesis, smooth muscle hypertrophy, and hyperplasia were up-regulated significantly in rat bladder tissue as a result of SCI [10]. Of special interest in this up-regulated set of transcripts were tropoelastin, lysyl oxidase, IGF-1, and TGF-β1, which were all significantly up-regulated as compared to normal rat bladder at all three time points post-SCI. The goal of this study was to identify mechanistic targets in the form of pathways modulated by SCI; further work in additional animals is needed in order to establish the role of these pathways in the pathology observed in this animal model. The present pathways analysis corroborates the original microarray results [10] on the significance of remodeling in bladder tissue after SCI, and it reveals a greater role for local and systemic inflammation than had been previously appreciated in this setting. Additionally, our study suggests that the alarm/danger response [53] may play an early, central role post-SCI. We suggest that the coupled use of DNA microarrays and pathways analysis techniques will help define molecular milestones associated with the initiation, development, and progression of bladder complications accompanying disorders such as SCI.

## Supporting Information

Table S1 Focus genes and top functions resulting from the network analysis, per network, at 3 days post-SCI.
(0.02 MB PDF)

Table S2 Focus genes and top functions resulting from the network analysis, per network, at 7 days post-SCI
(0.01 MB PDF)

Table S3 Focus genes and top functions resulting from the network analysis, per network, at 25 days post-SCI
(0.02 MB PDF)

Figure S1 Merged view of the first five networks for three days post-SCI. Arrows indicate the most relevant genes. An asterisk (*) indicates that a given gene is represented in the microarray set with multiple identifiers. The gene numbers are also listed in Table 1–2.
(1.41 MB TIF)

Figure S2 view of the first six networks for ten days post-SCI. Arrows indicate the most relevant genes. An asterisk (*) indicates that a given gene is represented in the microarray set with multiple identifiers. The gene numbers are also listed in Table 1–2.
(2.17 MB TIF)

Figure S3 Merged view of the first seven networks for 25 days post-SCI. Arrows indicate the most relevant genes. An asterisk (*) indicates that a given gene is represented in the microarray set with multiple identifiers. The gene numbers are also listed in Table 1–2.
(2.44 MB TIF)

Figure S4 Functional analysis of the complete data set. The most significant and relevant functions are listed for each time point post-SCI. The negative value of the log of the p-value is plotted for each function. A) Three days post-SCI. B) Ten days post-SCI. C) 25 days post-SCI.
(0.12 MB PDF)

Figure S5 Significant canonical pathways for each time point post-SCI. The negative value of the log of the p-value is plotted for each pathway, together with the signal log ratio value (ratio). A) Three days post-SCI. B) Ten days post-SCI. C) 25 days post-SCI.
(0.13 MB PDF)
