# Supplementary material for: An Exploratory Pathways Analysis of Temporal Changes Induced by Spinal Cord Injury in the Rat Bladder Wall: Insights on Remodeling and Inflammation
Source: PLoS One. 2009 Jun 9;4(6):e5852. doi: 10.1371/journal.pone.0005852 (PMC2688838; doi:10.1371/journal.pone.0005852)
Supplement: Table S1 — Focus genes and top functions resulting from the network analysis, per network, at 3 days post-SCI. (0.02 MB PDF) [file pone.0005852.s001.pdf]

SUPPLEMENTARY TABLES

Table S1. Focus genes and top functions resulting from the network analysis, per network, at 3 days post-SCI.

No.[#]	Molecules in Network			Score	Focus genes	Top functions
	Up-regulated	Down-regulated	IPKB genes not in data set			
1	ACPP, ACTA1, ACTC1*, COL5A1, CORO1A, CSRP2, **ELN**, ENO1, KRT5, KRT14, KRT18, **LOX***, LOXL1*, SLC7A1, **TGFB1**, TPM3	ADH1C (includes EG:126), CALD1, CAP2, CNN3, **DBP***, EEF1A1, EPB41, MS4A2, NEXN, P4HA1, PER2, PRKCB1, TNNT2, TPM1*	Actin, Actin-&alpha, F Actin, Pak, Tropomyosin	48	30	Cellular Assembly and Organization, Dermatological Diseases and Conditions, Genetic Disorder
2	CCL13*, CFD, **DUSP1***, FDPS*, FST, GCH1, PENK, PTPN1, TRH	AGTR2, ATP5E, CD59*, **GHR**, GNAI3, GNAO1, GNAQ, INSR, KCNA5, KLF9, NMBR, NR1D1*, NR1D2, P2RY1, PIK3R1, PRLR, SLC2A4, **THRB**, TSHB	Adenylate Cyclase, C8, G alpha, G alphai, G-protein beta, Mapk, PI3K	43	28	Carbohydrate Metabolism, Endocrine System Disorders, Molecular Transport
3	ATF3, **CD74***, ERP29, F3, FCGR2A*, FLT1, HLA-DMA, HLA-DMB, **HLA-DQB2***, HLA-DRA*, HRAS, LGALS7, SC4MOL, SDC1, SQLE, SYK, TGFB3	ANK3*, CCL11, CD9, GRIN3A, ITGA1, JUND, MAOB, NPTN*, NR2F1, PTPRS (includes EG:5802), SMC3	Ap1, Fgf, MHC Class II, MHC II-&beta, NMDA Receptor, PEPCK, PLC gamma	43	28	Immune Response, Cell-To-Cell Signaling and Interaction, Immune and Lymphatic System Development and Function
4	CAT*, CYR61, FGG, FN1*, IFRD1, **IGF1**, MMP14, MMP23B, PLAT, **SERPINE1**, SPP1, **THY1**, TIMP1*	ATP2B4, DCN, IGFALS, IGFBP2, **IGFBP3***, IGFBP5, MMP11, MYH11, PLP1, PPP3CA, PPP3CB, SFRS3, TGFB2, UBE2G1	Calcineurin A, Calcineurin protein(s), Ets, Fibrin, Igfbp, Irs, Mmp, Tgf beta	41	27	Tissue Development, Cellular Movement, Skeletal and Muscular System Development and Function
5	ADCY5, KCNC2, LYZ, MYC, RBP1, SLC2A2	AKAP1, ALCAM, ATP2A2*, BMI1, CAMK2D, CAST, CNGB1, CSDE1*, LOC289233, PCP4, PDE1B, PDE4B, PLN, PRKACB, RFC1, **RIMS1**, SNAP25, SYT1, TXNIP*, UGT1A6	Adaptor protein 2, Calmodulin, Camk, CaMKII, Pde, Pka, Pkac, PP1, SERCA,	39	26	Cellular Assembly and Organization, Cellular Function and Maintenance, Cardiovascular System Development and Function
6	BDNF, CCNB1, CDC2, CDC20, DGCR6L, JUNB, NR4A1, PRKCH, PVR, RGC32, TOP2A	ALDH3A1, CCND2, CHRNA4, DMTF1, GNAZ, ID2, ITPR1, MPDZ, NTF3, NTRK3, PKN2, RB1, SMARCA2, TMPO	Akt, Cyclin B, Cyclin D, Cyclin E, E2f, Gsk3, Hdac, MEF2, Neurotrophin, Rb,	37	25	Cell Cycle, Cell Death, Neurological Disease
7	ACAT2, ALDH1A2, APOE*, BMP3, CEBPB, **CEBPD***, CYP51A1, EGR2, NEUROG1, OLR1 (includes EG:4973), PLAUR, SMAD1, UCHL1*	APOB, AR, CSNK2A1, EIF5*, GARNL1, NCL, PLCL1, SSB*, SVIL, ZFP386	Bmp, Cbp/p300, Ck2, EPPB9, HPR, N-cor, RNA polymerase II, RPS27A, SMYD3, Stat, TSG101, Ubiquitin	32	23	Gene Expression, Embryonic Development, Protein Synthesis

[#] Only those networks are depicted that received the highest scores in IPA and that contained relevant genes.
* Gene is represented in the microarray set with multiple identifiers.
Bold: Gene was analyzed and discussed in more detail.
