# Supplementary material for: An Exploratory Pathways Analysis of Temporal Changes Induced by Spinal Cord Injury in the Rat Bladder Wall: Insights on Remodeling and Inflammation
Source: PLoS One. 2009 Jun 9;4(6):e5852. doi: 10.1371/journal.pone.0005852 (PMC2688838; doi:10.1371/journal.pone.0005852)
Supplement: Table S2 — Focus genes and top functions resulting from the network analysis, per network, at 7 days post-SCI (0.01 MB PDF) [file pone.0005852.s002.pdf]

Table S2. Focus genes and top functions resulting from the network analysis, per network, at 7 days post-SCI.

No.	Up-regulated genes in network	Down-regulated genes in network	IPKB genes not in data set	Score	Focus genes	Top functions
1	CDC20, **ELN**, ENO1, FABP5, FTS, GREM1, HMGB2*, IGF2R, KRT8, KRT19*, **LOX**, LYZ, OCLN*, SLC7A1, TGFB1, TGFBR3, TPM1, TRPC1	ANP32A, CALD1, CUGBP2, DDR1, EIF4H, JAG2, PACS1, PDPK1, PGCP, PSMD1, SLC5A5	Ctbp, Fgfr, p70 S6k, Pla2, PP2A, RTK	49	29	Embryonic Development, Tissue Morphology, Dermatological Diseases and Conditions
2	A2M, AIF1, APOE, **GHR**, HK2, IGFBP2*, IL1B, MMP8, PLAT, **S100A8**, **S100A9**, TIMP1*, UGCG	AGTR1B, APBB1, CYB5R3, **DBP***, ENPP2*, FAM105B, IFNGR1, **IGFBP3***, KCNH2, LDLR, NR1D1, NR1D2, NR4A3, PPP1R12A, PRLR	Fibrin, Igfbp, IL1, Jak, Mmp, Pdgf, Stat	47	28	Hematological System Development and Function, Tissue Morphology, Cellular Movement
3	ADCYAP1, AURKB, CCNB1, **CD74***, CEBPB, **CEBPD**, DUSP6, HLA-DRA, MCM6, RB1, SDC1, TOP2A*	ACAT1, CYP11B1, ID2, **IL4**, ITGA1, KCNC2, LDB3, NR2F1, **PRDM2**, RALBP1, S100G, SMC1A	14-3-3, Cyclin B, Cyclin E, E2f, Histone h3, MHC Class II, P38 MAPK, PEPCK, Pka, Rb, RNA polymerase II	37	24	Cellular Growth and Proliferation, Skeletal and Muscular System Development and Function, Cell Cycle
4	BAX*, CA2, CORO1A, FCGR2A*, FDPS, FN1*, KALRN, KLRD1, PTPN6, **SERPINE1**, SPP1, STAT1, STMN1, TAP1, TFRC	ACHE, ACTB, AGTR1, CAP2, GOT2, **THRB**, WIPF3	Actin, Caspase, Cbp/p300, F Actin, Gi-coupled receptor, Hsp70, Jnk, Mapk, MHC Class I, Pak, Rac, Tgf beta, Tnf receptor,	33	22	Cell Death, Organismal Injury and Abnormalities, Immunological Disease
5	HCK, **IGF1**, PTPN11, RASGRP1, SLC2A1, SYK, **THY1**	ATP2A1, ATP2B1, CHRM2, GNAO1, GNAS, GNAZ, GNB5, IRS2, ITPR1, PDE4B, PLCL1, PLN	Adenylate Cyclase, CD3, G alpha, G alphai, G-protein beta, G-protein gamma, Hdac, Irs, Pde, PI3K, Rap1, Ras, SERCA, Sos, TCR, Vav	27	19	Cell Signaling, Molecular Transport, Vitamin and Mineral Metabolism
6	FLT1, HMOX1, MFGE8, SERPINB5	CENTA1, CYP3A4, GABBR2, GRIN1, GRIN2B, GRIN2C, GRIN2D, GRM7, HD (includes EG:3064), MAG (includes EG:4099), MAP2K5, NEFL (includes EG:4747), NEFM, **PLA2G2A**, PPAP2B	Akt, Ap1, Calcineurin protein(s), Calmodulin, CaMKII, Cpla2, Creb, Ets, Fgf, GRIN, Hsp90, NMDA Receptor, Pkc(s), PLC gamma, Rsk, Vegf	27	19	Neurological Disease, Organismal Injury and Abnormalities, Respiratory Disease

[#] Only those networks are depicted that received the highest scores in IPA and that contained relevant genes.
* Gene is represented in the microarray set with multiple identifiers.
Bold: Gene was analyzed and discussed in more detail
