# Supplementary figures and images for: An Exploratory Pathways Analysis of Temporal Changes Induced by Spinal Cord Injury in the Rat Bladder Wall: Insights on Remodeling and Inflammation
Source: PLoS One. 2009 Jun 9;4(6):e5852. doi: 10.1371/journal.pone.0005852 (PMC2688838; doi:10.1371/journal.pone.0005852)

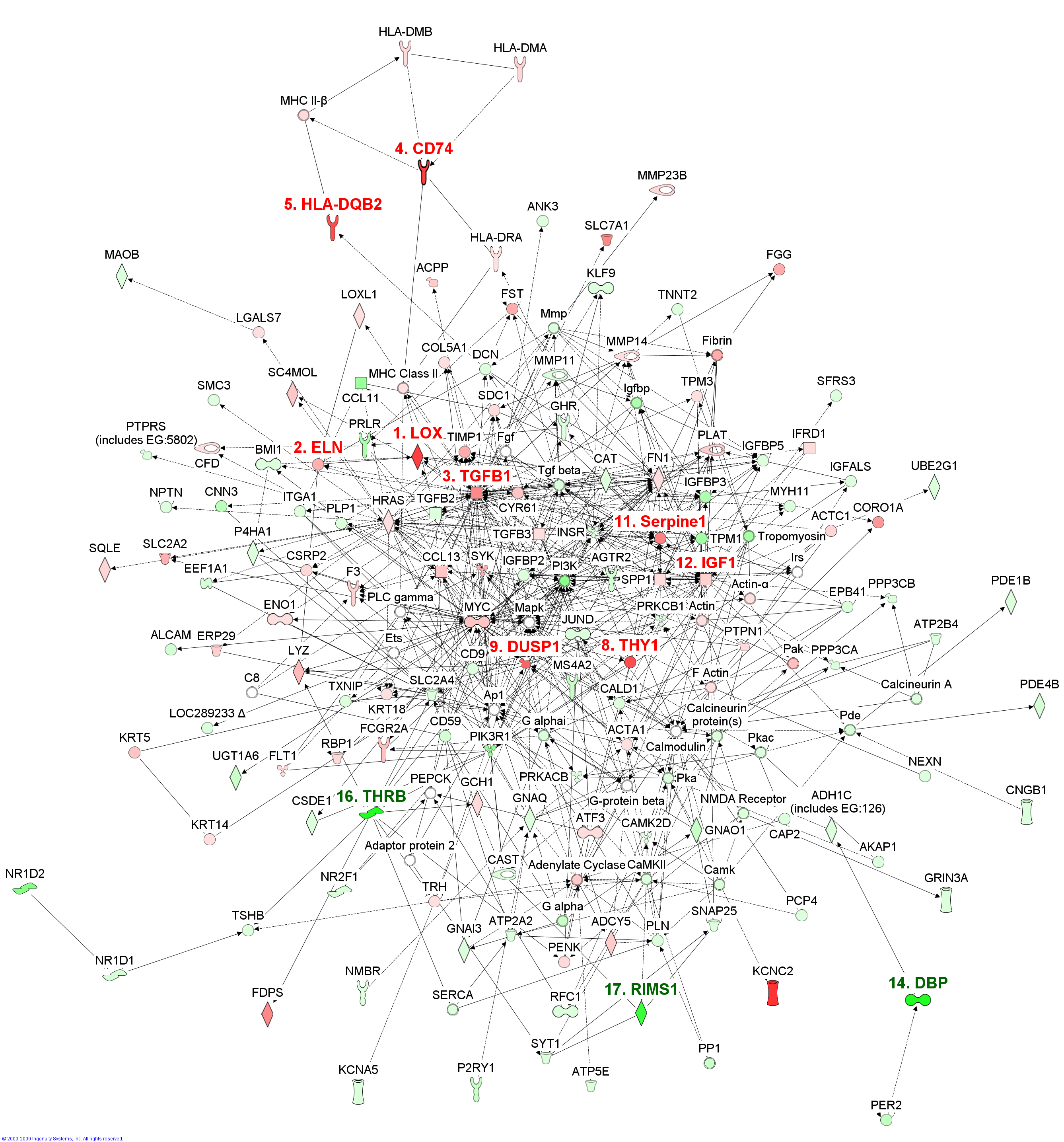

Supplement: Figure S1 — Merged view of the first five networks for three days post-SCI. Arrows indicate the most relevant genes. An asterisk (*) indicates that a given gene is represented in the microarray set with multiple identifiers. The gene numbers are also listed in Table 1–2. (1.41 MB TIF) [file pone.0005852.s004.tif]

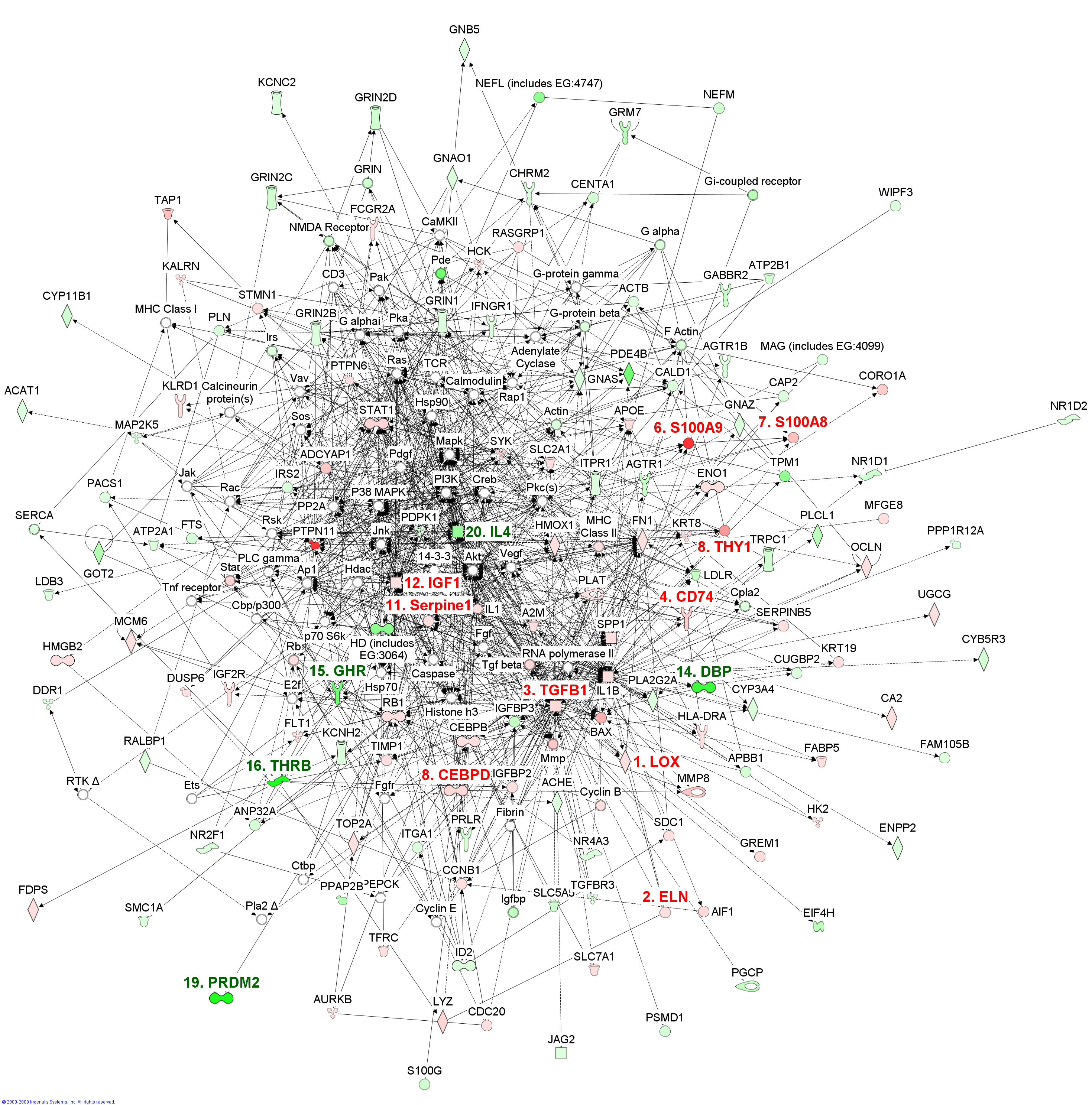

Supplement: Figure S2 — view of the first six networks for ten days post-SCI. Arrows indicate the most relevant genes. An asterisk (*) indicates that a given gene is represented in the microarray set with multiple identifiers. The gene numbers are also listed in Table 1–2. (2.17 MB TIF) [file pone.0005852.s005.tif]

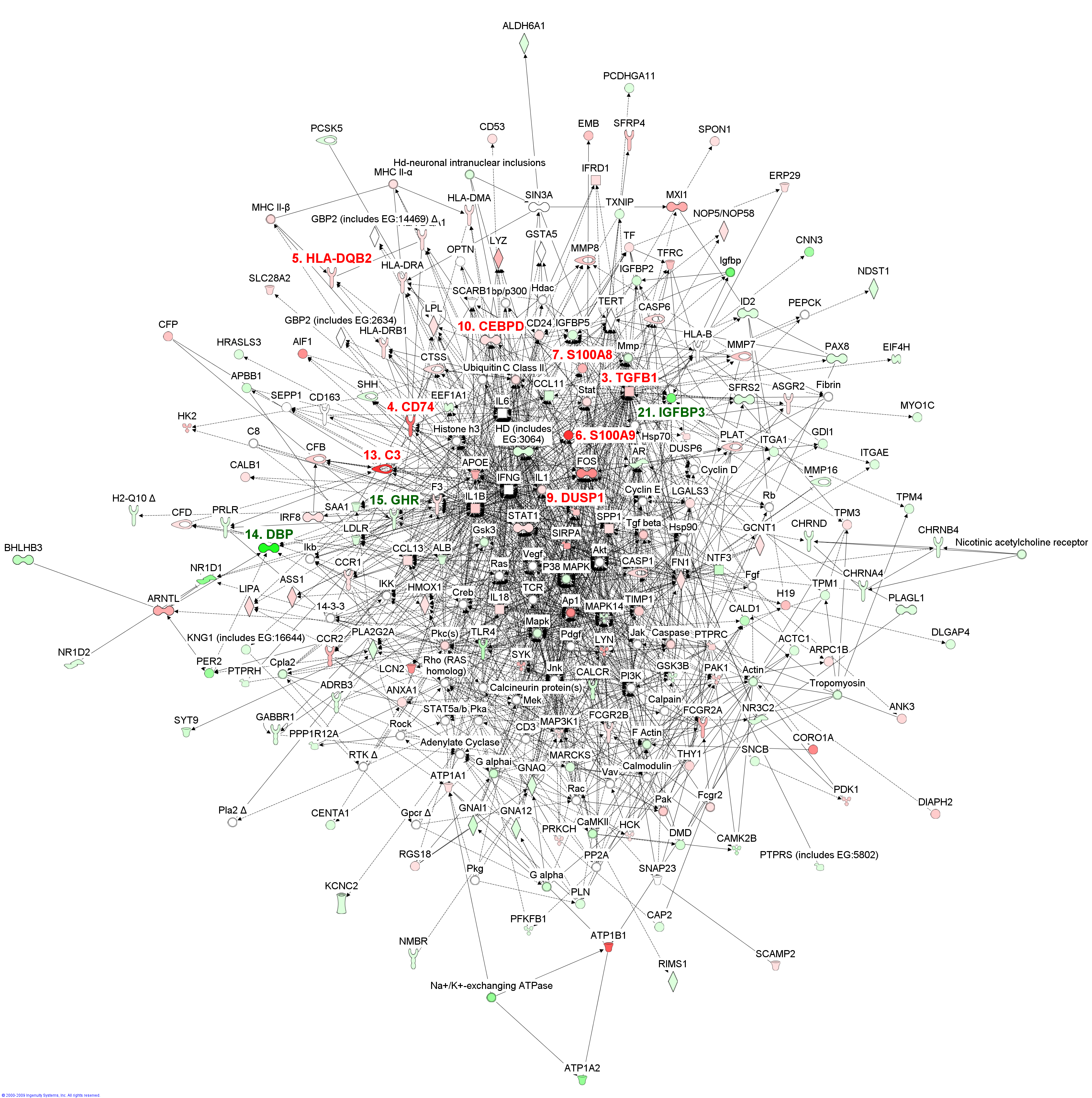

Supplement: Figure S3 — Merged view of the first seven networks for 25 days post-SCI. Arrows indicate the most relevant genes. An asterisk (*) indicates that a given gene is represented in the microarray set with multiple identifiers. The gene numbers are also listed in Table 1–2. (2.44 MB TIF) [file pone.0005852.s006.tif]

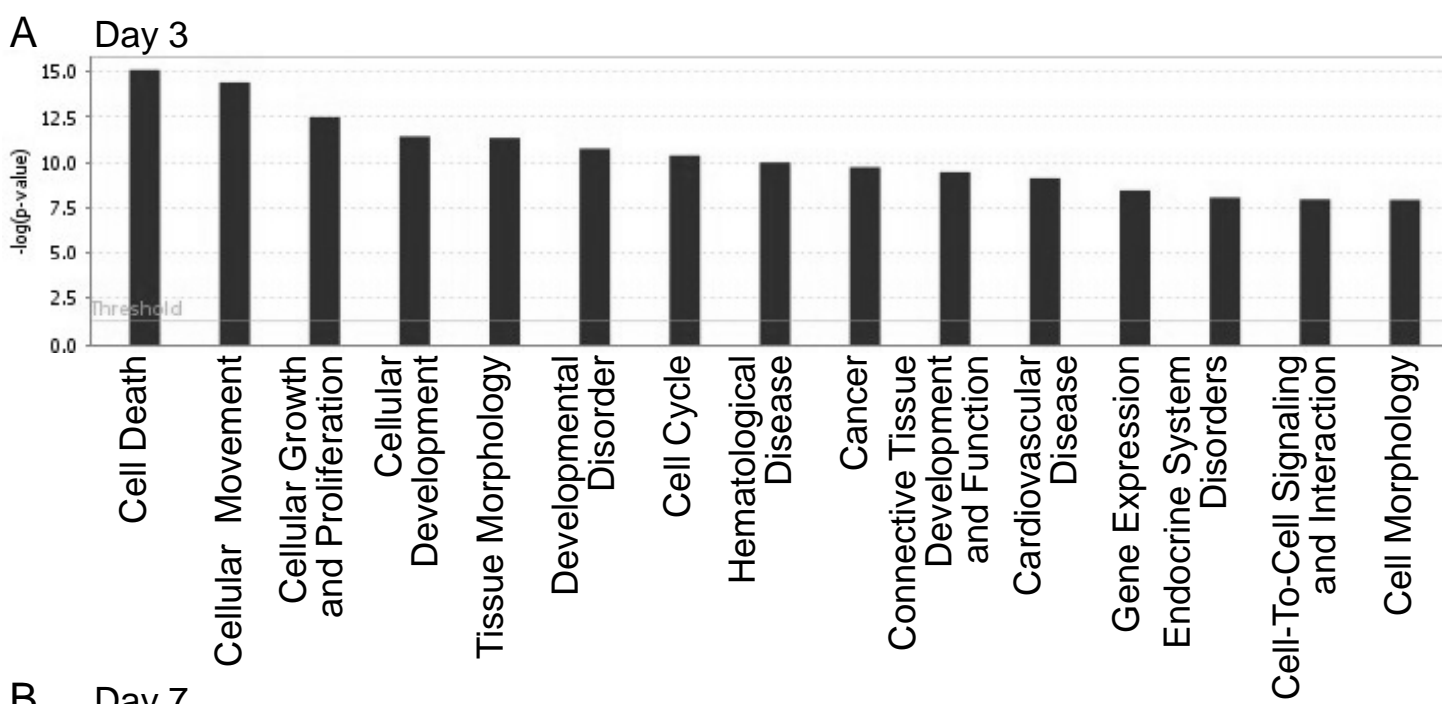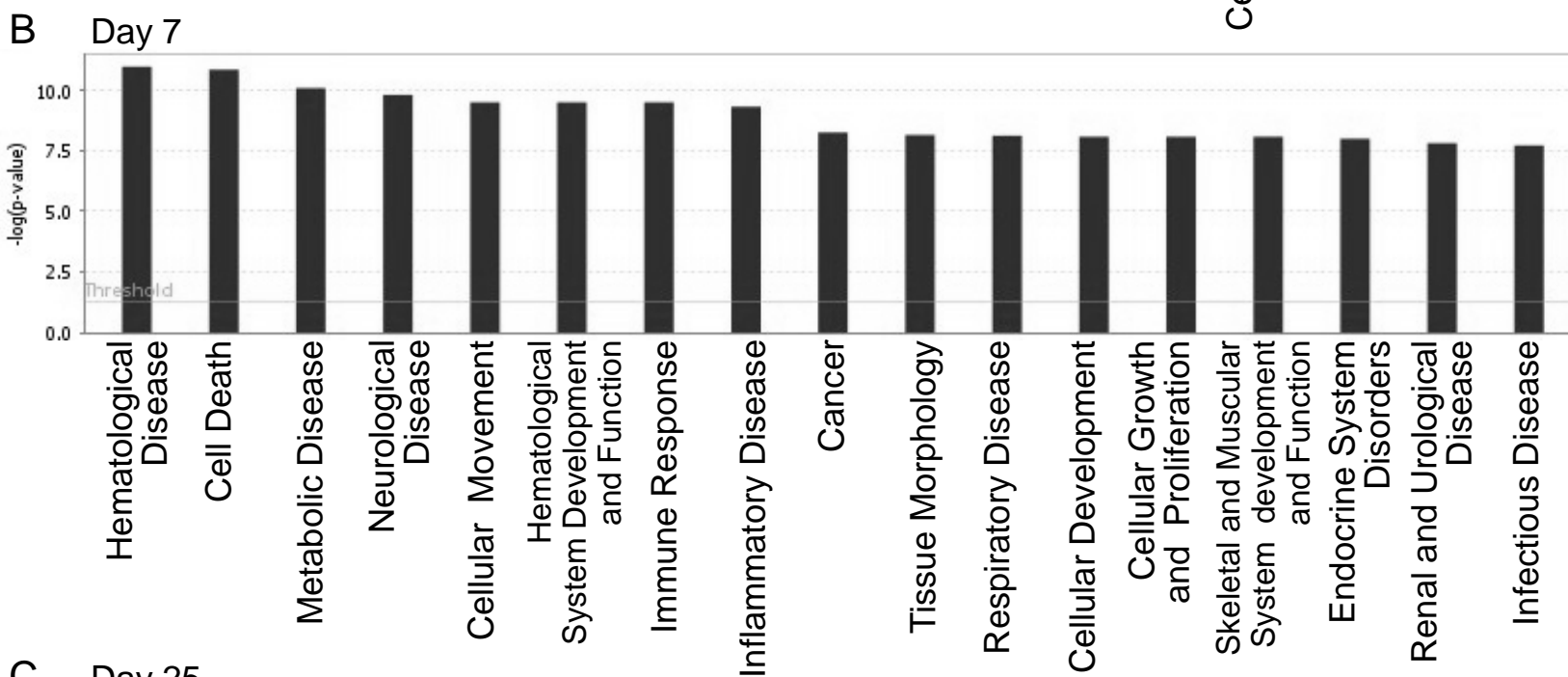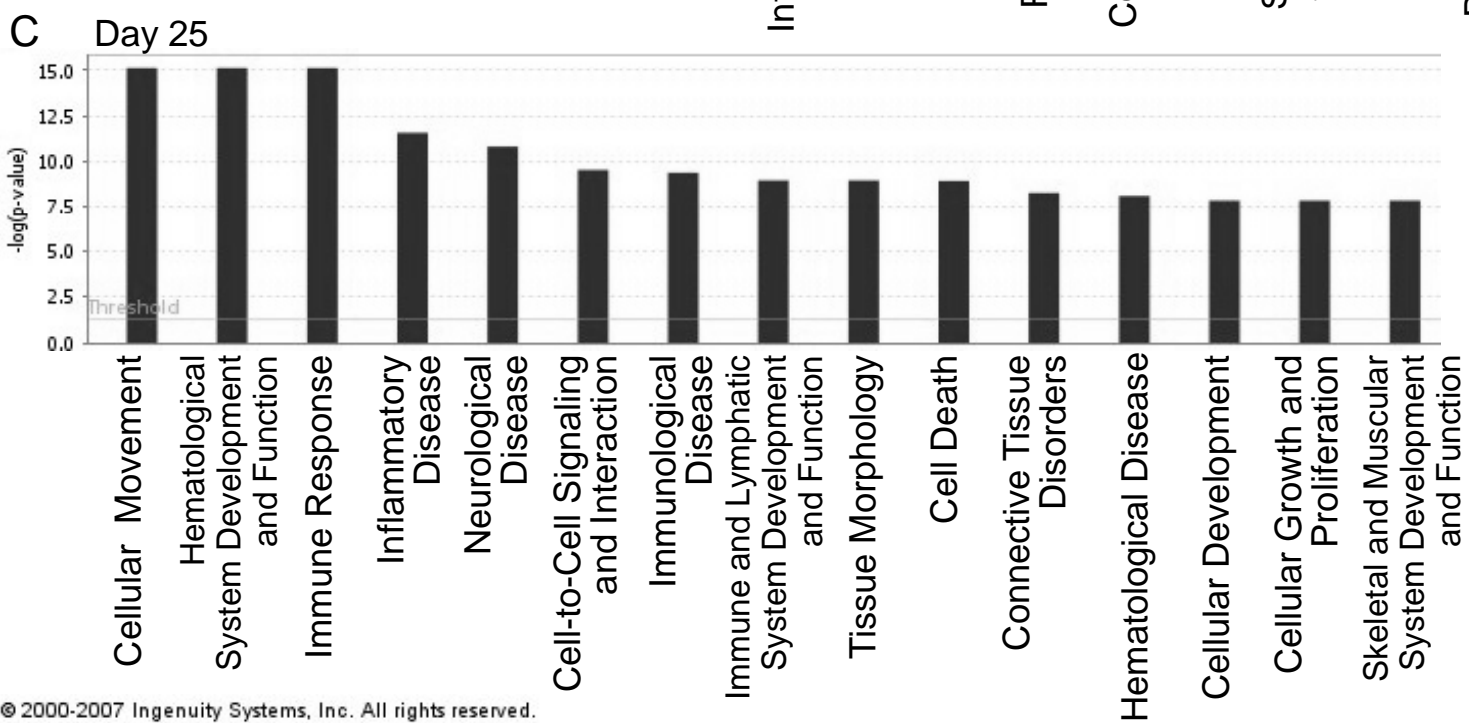

Supplement: Figure S4 — Functional analysis of the complete data set. The most significant and relevant functions are listed for each time point post-SCI. The negative value of the log of the p-value is plotted for each function. A) Three days post-SCI. B) Ten days post-SCI. C) 25 days post-SCI. (0.12 MB PDF) [file pone.0005852.s007.pdf]

A Day 3

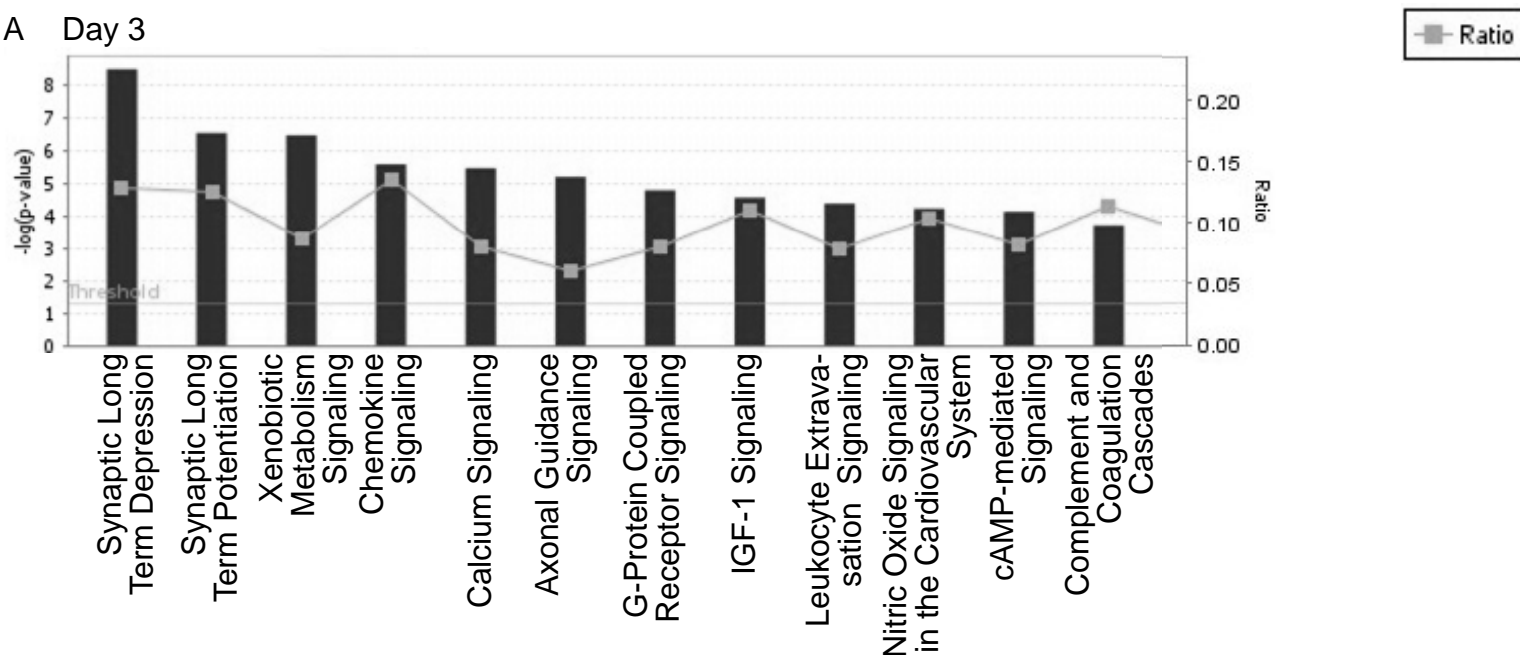

B Day 7

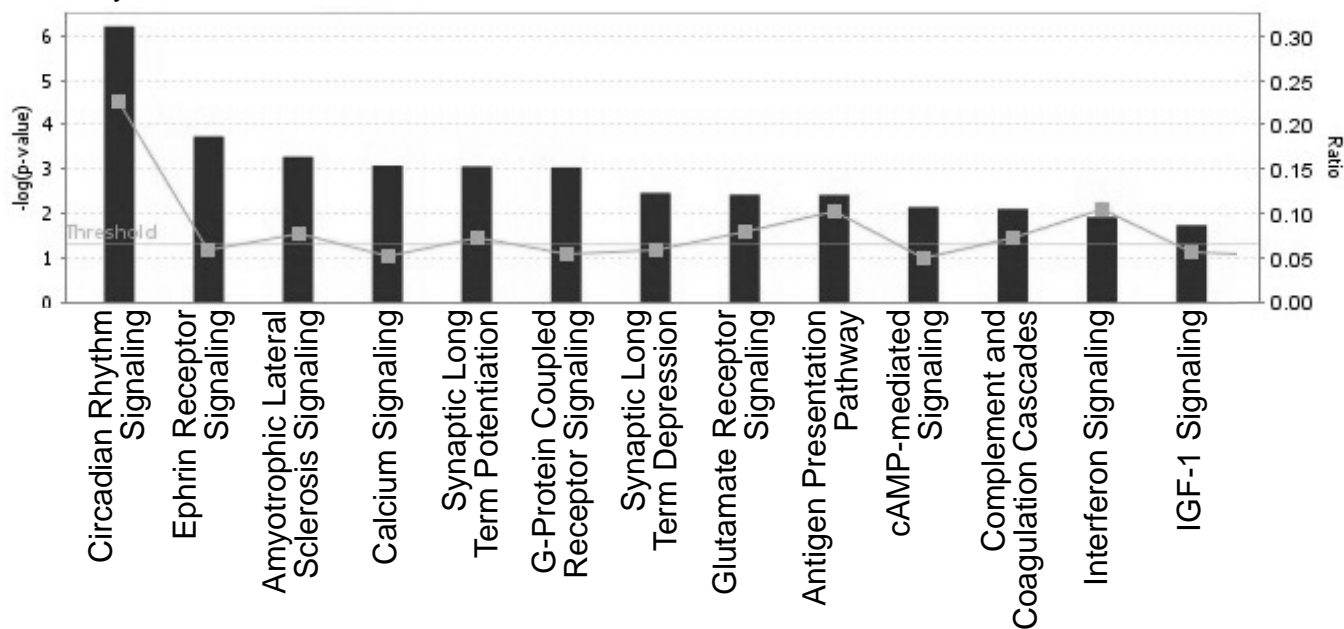

C Day 25

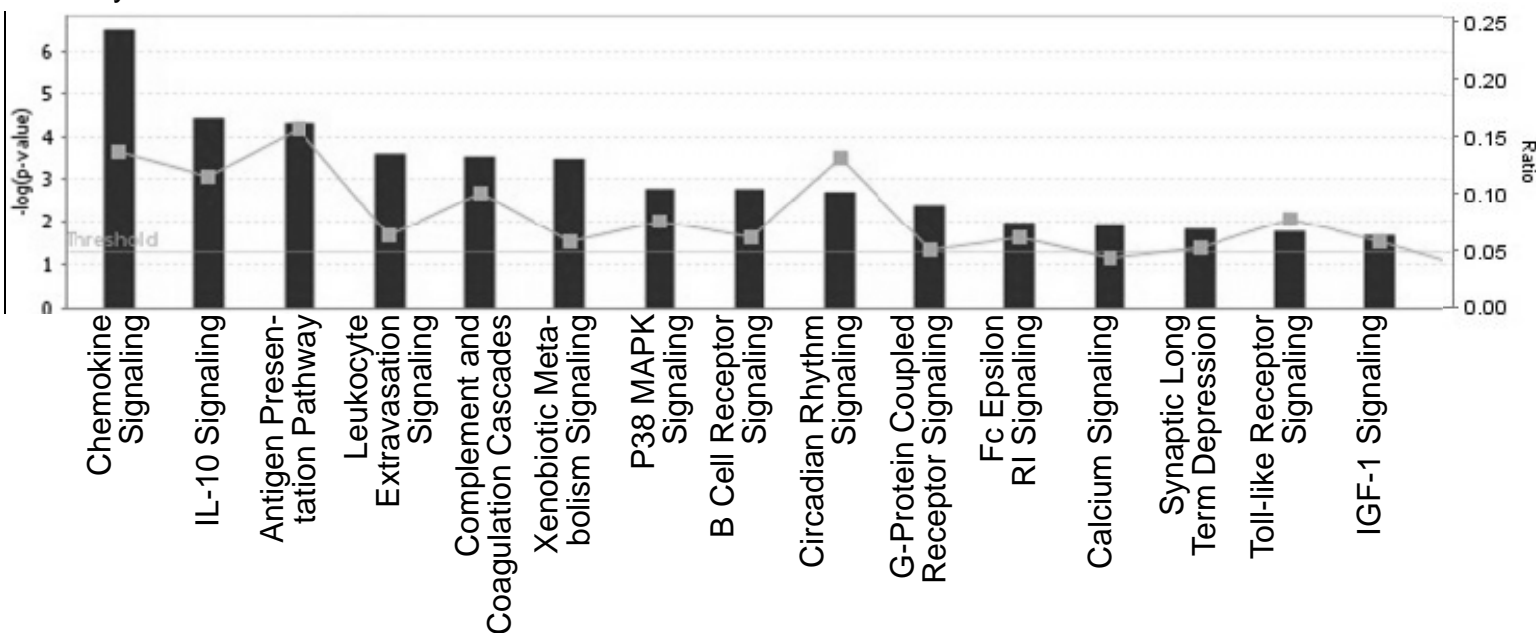

Supplement: Figure S5 — Significant canonical pathways for each time point post-SCI. The negative value of the log of the p-value is plotted for each pathway, together with the signal log ratio value (ratio). A) Three days post-SCI. B) Ten days post-SCI. C) 25 days post-SCI. (0.13 MB PDF) [file pone.0005852.s008.pdf]
