# Supplementary material for: An Exploratory Pathways Analysis of Temporal Changes Induced by Spinal Cord Injury in the Rat Bladder Wall: Insights on Remodeling and Inflammation
Source: PLoS One. 2009 Jun 9;4(6):e5852. doi: 10.1371/journal.pone.0005852 (PMC2688838; doi:10.1371/journal.pone.0005852)
Supplement: Table S3 — Focus genes and top functions resulting from the network analysis, per network, at 25 days post-SCI (0.02 MB PDF) [file pone.0005852.s003.pdf]

Table S3. Focus genes and top functions resulting from the network analysis, per network, at 25 days post-SCI.

No.	Up-regulated genes in network	Down-regulated genes in network	IPKB genes not in data set	Score	Focus genes	Top functions
1	ANK3, ASGR2, CASP1, DIAPH2, GCNT1, HCK, MXI1, NOP5/NOP58, PAK1, PDK1, PTPRC, **TGFB1**, TPM3	ACTC1, CALD1, CAMK2B, CAP2, CNN3, EEF1A1, EIF4H, ID2, ITGAE, MYO1C, NDST1, PAX8, PTPRS (includes EG:5802), SNCB, TPM1*, TPM4, TXNIP	Actin, Jak, Pak, Tropomyosin, Vav	52	30	Carbohydrate Metabolism, Small Molecule Biochemistry, Cellular Function and Maintenance
2	AIF1*, ARNTL*, ASS1, CALB1, CCL13, CCR1, CCR2, CD24, **CD74***, CTSS, F3, H19*, HLA-DMA, HLA-DQA1*, **HLA-DQB2**, HLA-DRA*, HLA-DRB1*, IL1B*, LCN2, RGS18	APBB1, BHLHB3, **DBP***, H2-Q10*, LDLR, NR1D1*, NR1D2, PER2, TLR4	C8, G alphai, Ikb, MHC Class II, MHC II-&alpha, MHC II-&beta,	49	29	Immune Response, Immunological Disease, Neurological Disease
3	ARPC1B*, **CEBPD**, CORO1A, EMB, FN1, FOS, LGALS3, MMP7, MMP8, PLAT, SFRP4, SPP1, **THY1**, TIMP1*	ALB, DLGAP4, **GHR***, GSK3B, IGFBP2, **IGFBP3**, IGFBP5, MMP16, NTF3*	Akt, Cyclin D, Fgf, Fibrin, Gsk3, Igfbp, IL1, Mmp, Pdgf, PI3K, Stat, Tgf beta	35	23	Cellular Movement, Skeletal and Muscular System Development and Function, Cellular Growth and Proliferation
4	APOE*, **C3**, CASP6, CFB, CFP, ERP29, HK2, IFRD1, IRF8, LPL*, LYN, SIRPA, STAT1, SYK, TF*, TFRC	AR, KCNC2, NR3C2, PCSK5, PFKFB1, PLAGL1, SHH	Calmodulin, Caspase, Cbp/p300, Cyclin E, Hdac, Hsp70, Hsp90, Pka, PP2A, Rb, STAT5a/b, Ubiquitin	35	23	Cellular Function and Maintenance, Immune and Lymphatic System Development and Function, Organismal Development
5	ANXA1, ATP1A1, ATP1B1*, CENTA1, DUSP6, FCGR2A*, FCGR2B, HMOX1*, LIPA, MAP3K1, PRKCH, **S100A8**, **S100A9**	ADRB3, ATP1A2, GDI1, MARCKS, **PLA2G2A**, PLN, SAA1	CD3, Cpla2, F Actin, Fcgr2, IKK, Mek, Na+/K+-exchanging ATPase, P38 MAPK, PEPCK, Pkc(s), Pkg, Pla2, Ras, RTK, TCR	29	20	Cardiovascular System Development and Function, Organ Morphology, Cancer
6	CFD, **DUSP1**, IL18*, LYZ,	CALCR, CCL11, DMD, GABBR1, GNA12, GNAI1, GNAQ, ITGA1, KNG1 (includes EG:16644), MAPK14, NMBR, PPP1R12A, PRLR*, PTPRH, **RIMS1**, SYT9	14-3-3, Adenylate Cyclase, Ap1, Calcineurin protein(s), CaMKII, Creb, G alpha, Gpcr, Histone h3, Jnk, Mapk, Rac, Rho (RAS homolog), Rock, Vegf	29	20	Cardiac Hypertrophy, Cardiovascular Disease, Cellular Development
7	CD24, CD53, **CD74***, CTSS, HLA-DMA, HLA-DQA1*, **HLA-DQB2***, HLA-DRB1*, SCAMP2, SLC28A2, SPON1	ALDH6A1, CHRNA4, CHRNB4, CHRND, HD (includes EG:3064), HRASLS3, PCDHGA11, SFRS2	Calpain, CD163, GBP2 (includes EG:14469), GBP2 (includes EG:2634), GSTA5, Hd-neuronal intranuclear inclusions, HLA-B, IFNG, IL6, Nicotinic acetylcholine receptor, OPTN, SCARB1, SEPP1, SIN3A, SNAP23, TERT	27	19	Immune Response, Inflammatory Disease, Immunological Disease

# Only those networks are depicted that received the highest scores in IPA and that contained relevant genes.
* Gene is represented in the microarray set with multiple identifiers;
Bold: Gene was analyzed and discussed in more detail
